# The ENDpoiNTs Project: Novel Testing Strategies for Endocrine Disruptors Linked to Developmental Neurotoxicity

**DOI:** 10.3390/ijms21113978

**Published:** 2020-06-01

**Authors:** Diana Lupu, Patrik Andersson, Carl-Gustaf Bornehag, Barbara Demeneix, Ellen Fritsche, Chris Gennings, Walter Lichtensteiger, Marcel Leist, Pim E. G. Leonards, Anne-Louise Ponsonby, Martin Scholze, Giuseppe Testa, Jesus A. F. Tresguerres, Remco H. S. Westerink, Bernard Zalc, Joëlle Rüegg

**Affiliations:** 1 Evolutionary Biology Centre, Uppsala University, 75236 Uppsala, Sweden; joelle.ruegg@ebc.uu.se; 2 Institute of Environmental Medicine, Karolinska Institute, 17177 Stockholm, Sweden; 3 Department of Toxicology, Faculty of Pharmacy, Iuliu Hatieganu University of Medicine and Pharmacy, 400012 Cluj-Napoca, Romania; 4 Faculty of Science and Technology, Umeå University, 90187 Umeå, Sweden; patrik.andersson@umu.se; 5 Department of Health Sciences, Karlstad University, 65188 Karlstad, Sweden; carl-gustaf.bornehag@kau.se; 6 Evolution of Endocrine Regulations UMR 7221, Centre National de la Recherche Scientifique, 75005 Paris, France; demeneix@mnhn.fr; 7 IUF—Leibniz Research Institute for Environmental Medicine, 40225 Düsseldorf, Germany; ellen.fritsche@iuf-duesseldorf.de; 8 Department of Environmental Medicine and Public Health, Icahn School of Medicine at Mount Sinai, New York, NY 10029, USA; chris.gennings@mssm.edu; 9 GREEN Tox GmbH, CH-8057 Zürich, Switzerland; walter.lichtensteiger@access.uzh.ch; 10 In Vitro Toxicology and Biomedicine, University of Konstanz, D-78457 Konstanz, Germany; marcel.leist@uni-konstanz.de; 11 Department Environment and Health, Vrije University, 1081HV Amsterdam, The Netherlands; pim.leonards@vu.nl; 12 Murdoch Children’s Research Institute, Royal Children’s Hospital, Parkville, Victoria 3052, Australia; annelouise.ponsonby@florey.edu.au; 13 Institute of Environment, Health and Societies, Brunel University London, Uxbridge UB8 3PH, UK; martin.scholze@brunel.ac.uk; 14 Department of Oncology and Hemato-Oncology, University of Milan, 20122 Milan, Italy; Giuseppe.Testa@unimi.it; 15 Department of Physiology, School of Medicine, Complutense University of Madrid, 28040 Madrid, Spain; guerres@ucm.es; 16 Institute for Risk Assessment Sciences (IRAS), Faculty of Veterinary Medicine, Utrecht University, 3584 CM Utrecht, The Netherlands; r.westerink@uu.nl; 17 Sorbonne Université, Inserm, CNRS, ICM-GH Pitié-Salpêtrière, 75651 Paris, France; bernard.zalc@upmc.fr

**Keywords:** endocrine-disrupting chemicals, developmental neurotoxicity, chemical testing, adverse outcome pathways

## Abstract

Ubiquitous exposure to endocrine-disrupting chemicals (EDCs) has caused serious concerns about the ability of these chemicals to affect neurodevelopment, among others. Since endocrine disruption (ED)-induced developmental neurotoxicity (DNT) is hardly covered by the chemical testing tools that are currently in regulatory use, the Horizon 2020 research and innovation action ENDpoiNTs has been launched to fill the scientific and methodological gaps related to the assessment of this type of chemical toxicity. The ENDpoiNTs project will generate new knowledge about ED-induced DNT and aims to develop and improve in vitro, in vivo, and in silico models pertaining to ED-linked DNT outcomes for chemical testing. This will be achieved by establishing correlative and causal links between known and novel neurodevelopmental endpoints and endocrine pathways through integration of molecular, cellular, and organismal data from in vitro and in vivo models. Based on this knowledge, the project aims to provide adverse outcome pathways (AOPs) for ED-induced DNT and to develop and integrate new testing tools with high relevance for human health into European and international regulatory frameworks.

## 1. Introduction

Worldwide, serious concern has arisen about human exposure to manufactured chemicals that can undoubtedly produce adverse health effects through disruption of the body’s endocrine system, known as endocrine-disrupting chemicals (EDCs) [1,2]. Therefore, at present, there is an urgent need to refine the global regulatory requirements for EDCs by improving the evidence base, updating and harmonizing the current chemical screening and testing tools in conjunction with human exposure–disease studies.

A significant knowledge gap is how EDCs affect neurodevelopment, and endocrine disruption (ED)-induced developmental neurotoxicity (DNT) is hardly covered by the testing tools that are currently in regulatory use. The brain is among the most vulnerable organs with respect to toxic insults [3], in particular during development, and EDCs have indeed been shown to target the (developing) brain. In the past decade, human epidemiology has provided evidence for positive associations between pre- and postnatal exposure to certain chemicals, including known EDCs (e.g., PCBs, PBDEs, PFOS, bisphenol A, phthalates, paracetamol) and impaired neurodevelopmental outcomes in children. Data from birth cohorts have shown that chemical exposure during time periods that are critical for neurodevelopment can adversely impact cognitive functions (memory, language), attention, emotions, and social behaviors, partly with sexual dimorphic effects [4,5,6,7,8,9,10,11]. These observations are supported by experimental data in rodent models demonstrating persistent effects on behavior, cognition, and motor activity after exposure to EDCs during development, including PCBs, PBDEs, PFOS, bisphenol A, phthalates, and vinclozolin [12,13,14,15,16].

Yet, in the context of brain development, and hormonal involvement therein, there are considerable species differences [17,18]. For example, brain sexual differentiation (i.e., masculinization and feminization of sexual and non-sexual behaviors) is orchestrated by sex steroids during specific developmental windows with differences between species [19]. In primates, this process happens during mid to late gestation and is driven by androgens, whereas in rodents it takes place just before and after birth and is directed by estradiol [19]. Thus, due to species disparities in the neuroendocrine landscape during development, current animal-based testing methods by themselves are most likely neither specific nor sensitive enough to predict effects relevant for human health. Furthermore, chemical exposures do not occur in isolation, but mostly as chemical mixtures act in concert with other environmental agents on humans with differing genetic vulnerability.

**Figure 1:**
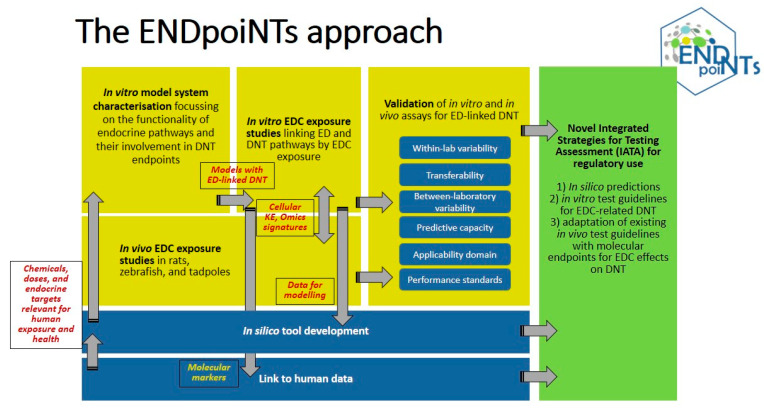
The methodological approach of ENDpoiNTs.

To address both the scientific and the methodological gaps, the Horizon 2020 research and innovation action ENDpoiNTs has been launched. ENDpoiNTs is part of the EURION cluster and includes 17 participants in Europe, USA, and Australia, integrating expertise in endocrine disruption and DNT and combines state-of-the-art in silico and in vitro tools, innovative experimental designs and technologies, and advanced biostatistics on human epidemiological and biomonitoring data. ENDpoiNTs will generate new knowledge about ED-induced DNT and aims to develop and improve in vitro, in vivo, and in silico models pertaining to ED-linked DNT outcomes for chemical testing (Figure 1). ENDpoiNTs will achieve this by establishing correlative and causal links between established and novel neurodevelopmental endpoints and endocrine pathways, including both well-studied EDC targets (such as the estrogen, androgen, and thyroid systems) and less-studied ones (such as the retinoic acid system).

The project will also generate knowledge about species and sex differences and similarities for ED-induced DNT by integrating molecular, cellular, and organismal data from female and male in vitro (both human and rodent primary and/or pluripotent cell-based) and in vivo models. Furthermore, it will link these insights to human health by the use of appropriate test compounds in concentrations relevant for human exposure and by linking back from the experimental results to human epidemiology data.

Based on this knowledge, the project aims to provide adverse outcome pathways (AOPs) for ED-induced DNT and to develop and integrate new testing tools with high relevance for human health into European and international regulatory frameworks.

## 2. Human-Relevant Exposure to EDCs

Widespread exposures to EDCs have been established by international biomonitoring programs (e.g., HBM4EU) and show regional and temporal variations. Moreover, during a lifetime, humans are clearly exposed to multiple EDCs in complex mixtures from various routes and sources, and these mixtures include multiple chemical classes. Most importantly, exposure to EDCs, in particular during early life, has been clearly associated with a number of adverse health outcomes in [2,4,5,6,7,8,9,10,11]. There is convincing evidence that early-life EDC exposure is associated with impairments in executive brain functions, speed of information processing, verbal abilities, visual recognition memory, increased externalizing behaviors, and lower IQ scores [7,8,9,10]. This has been shown for single EDCs, but recently also within a mixture approach [20]. Thus, it is crucial that we establish test systems that are sensitive enough to detect ED-related effects in the range of relevant human exposures and to detect effects from human-relevant mixtures of EDCs in addition to effects from single compounds.

The ENDpoiNTs project will include the establishment of human-relevant EDC exposure ranges from multiple international early life (pregnancy and postnatal) cohorts and biomonitoring studies. The primary pregnancy cohort is the Swedish SELMA study, which includes over 2300 pregnant women and their children, with prenatal exposure to 54 environmental chemicals and neurodevelopmental effects monitored in children up to 7 years of age. Other pregnancy cohorts with neuro-related outcomes included in the project are the Dutch LINC and the Australian Barwon Infant Study (BIS) cohorts. Additionally, ENDpoiNTs will make use of publicly available EU-wide biomonitoring data through HBM4EU and the US CHEAR studies.

The chemical compounds selected for testing within the project are based on previous results from the SELMA and the LINC cohorts in the H2020-funded EDC-MixRisk project and the FP7-funded DENAMIC project, respectively. It is a set of 27 known or suspected endocrine disruptors associated with human neurodevelopmental outcomes, such as cognition and behavior, in at least one of the projects, and comprises several pesticides, phthalates, bisphenols, perfluorinated compounds, and their key metabolites. The 27 parent compounds are listed in Table 1 and hereafter referred to as reference EDCs.

Both single EDCs and human-relevant mixtures of EDCs (i.e., with established human-relevant mixing proportions, concentration ranges, and associations to human DNT outcomes) will be tested.

## 3. Pathways Linking ED to DNT

The endocrine signaling pathways frequently studied in the context of ED (estrogen, androgen, thyroid, and to a lesser extent, glucocorticoid and retinoid signaling) play crucial roles in neurodevelopment and are therefore highly relevant for DNT-related endpoints [21]. However, the ENDpoiNTs project aims to broaden the spectrum of relevant pathways potentially contributing to ED-induced DNT. There are many more endocrine-related pathways involved in brain development that chemicals could interfere with and thus, there is a high probability that important effects may be overlooked. Using in silico predictions based on the results from the EDC-MixRisk and DENAMIC projects, we identified additional pathways associated with neurodevelopmental outcomes before the start of the ENDpoiNTs project (unpublished data). On top of the most studied targets in the context of EDCs, namely the estrogen receptors (ER), androgen receptors (AR), and thyroid receptors (TR), these analyses revealed pathways that have not or only to a small extent been studied in the context of endocrine disruption. All receptor targets intended for study are listed in Table 2 and their involvement in brain development is shortly summarized below.

One of the most thoroughly studied examples of hormonal programming of neurodevelopment is the role of sex steroids (estrogens and androgens) in the sexual differentiation of the brain. These hormones are known to be key drivers in the programming of sexual and socio-aggressive behaviors in males and females and in the development of sexually dimorphic, multifunctional brain structures such as the preoptic area, the ventromedial and arcuate nuclei of the hypothalamus, the bed nucleus of stria terminalis, and the amygdala [19,22,23,24,25]. As such, alterations in the early signaling through sex steroid pathways have been shown to result in alterations of reproductive development and behavior in laboratory animals [25]. Besides their role in the sexual differentiation of cerebral structures in the limbic system, sex steroids are also involved in corticogenesis and prenatal exposure to them has been linked to negative effects on cognition [26].

Thyroid hormone (TH) signaling is well-known for playing an essential role during brain development in all vertebrates [27,28]. In humans, maternal hypothyroidism during pregnancy is associated with decreased IQ and diminished performance on tests of fine motor skills, vocabulary, and speech in their children [29]. TH has been shown to affect oligodendrocyte commitment and maturation [30,31], and neuronal commitment [32] during mouse neurogenesis, as well as oligodendrocyte maturation in primary human neuronal precursor cells (NPCs) [31].

The glucocorticoid receptor (GR) is activated by its endogenous ligand cortisol and provides a vital negative feedback loop to modulate stress by downregulating the hypothalamic–pituitary–adrenal (HPA) axis [33]. Yet, GR activation during brain development inhibits the migration of post-mitotic neurons in rodents and decreases proliferation and cell survival in both rodent and human neuronal stem cells. In humans, high doses of a GR agonist during pregnancy leads to severe pathologies in children such as cognitive and motor disorders [34].

The retinoic acid and retinoic X receptors (RAR and RXR) are activated by the vitamin A metabolites all-trans and 9-cis retinoic acid (RA), respectively. RA signaling is essential for embryonic development and plays crucial roles in anteroposterior and dorsoventral patterning of the neural plate and neural tube. Furthermore, RA signaling controls proliferation and differentiation of neuronal stem cells into neurons, astrocytes, and oligodendrocytes [35].

Also, the other identified receptors, such as the progesterone receptor (PR), the peroxisome proliferator-activated receptors (PPARs), the vitamin D3 receptor (VDR), the liver X/oxysterols receptors (LXRs), and the prostaglandin receptors play important roles in the developing brain. Progesterone promotes the proliferation of neural progenitor cells and dopaminergic neuronal differentiation in the developing CNS. Furthermore, it is involved in brain sexual differentiation and has been shown to stimulate myelination not only through increasing myelin synthesis in oligodendrocytes, but also by inducing the proliferation and promoting the differentiation of oligodendrocyte progenitor cells [36,37]. PPAR gamma activation increases proliferation and counteracts apoptosis in human and rodent neuronal stem cells, and is involved in neuronal differentiation, while PPAR alpha and beta are implicated in the maturation of astrocytes and oligodendrocytes, respectively [35]. In the developing rodent brain, VDR is important for apoptotic processes and for the regulation of the expression of neurotrophic factors. Furthermore, it is implicated in neuronal differentiation and maturation, in particular of dopaminergic neurons [34]. In humans, vitamin D deficiency has been associated with schizophrenia, autism and depression [38]. **LXRs** have been shown to be involved in the differentiation of dopaminergic neurons [34], the migration of cortical neurons, and in the myelination process [39]. Finally, prostaglandin E2 (PGE2) has been shown to mediate the development of the medial preoptic area in rats, a critical region controlling male sexual behavior [40]. In humans, prenatal exposure to paracetamol, an inhibitor of prostaglandin synthetase in the brain, has been associated with adverse neurodevelopmental outcomes such as ADHD and autism spectrum disorder [41].

## 4. Relevant Models and Endpoints to Test ED-Induced DNT

In the EU and USA, identification of chemicals that have the potential to induce DNT is based on higher tiered animal testing. DNT testing is performed only rarely, upon evidence of neurotoxicity from acute or repeated dose toxicity studies in adult rodents, which is not always a reliable indicator for DNT [3,42,43,44]. Furthermore, in vitro tests for DNT are lacking in the entire OECD Guidelines Programme for the testing of chemicals. Implicitly, in vitro tests and in vivo endpoints for the assessment of ED-induced DNT are completely absent from chemical regulatory frameworks. Therefore, there is an urgent need to refine existing in vivo tests to capture ED-induced DNT and to develop testing methodologies suitable for the rapid and cost-effective identification of chemicals with DNT potential in general, and for chemicals with ED-linked DNT potential in particular (i.e., in vitro tests) [3,42,43,44].

The ENDpoiNTs battery of testing methods is designed to capture the essential endpoints of ED-induced DNT relevant for humans, covering molecular, cellular, and organismal key events (KE) and outcomes crucial for neurodevelopmental processes both in vitro and in vivo. The models are of different origins and complexities, and focus mainly on processes in the central nervous system (CNS), with two exceptions that address early events in peripheral nervous system development. Wherever applicable and feasible, the in vitro and in vivo endpoints are designed to interrogate effects on brain regions that are relevant in the context of EDC impact on human health.

**Figure 2:**
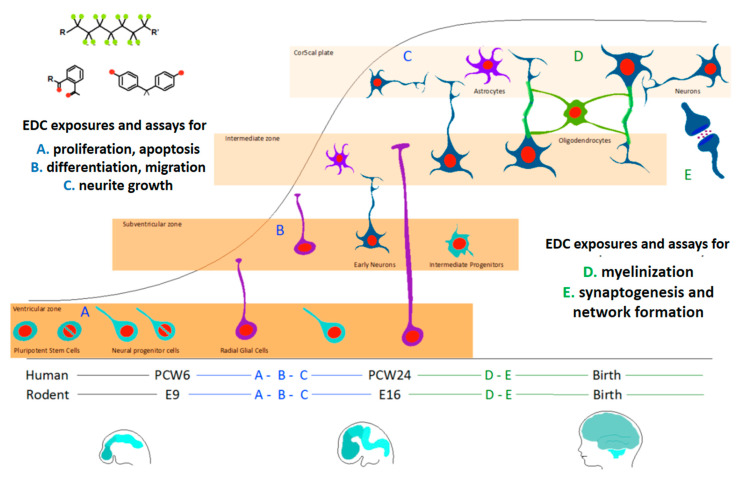
Key events during brain development which are addressed in the ENDpoiNTs assay battery. PCW: post-coital week; E: embryonic day. Figure adapted with permission [46].

The key events of brain development covered by the in vitro methods used in ENDpoiNTs have been chosen according to the available mechanistic insights pertaining to DNT (see Figure 2) [42,43,44,45]. The experimental models employed are summarized in Table 3.

The endpoints will be probed by cell biological (e.g., morphology, electric activity), biochemical (e.g., protein marker expression), transcriptomic (targeted and untargeted gene expression analyses), and epigenomic readouts, both already established and newly identified within this project. Since various genes encoded on the X and Y chromosomes are involved in brain development, and the sexual differentiation of the brain results from an interaction between the genetic background and the actions of sex steroid hormones [25,53,54], the in vitro models used will not only be of different species and cell origins, but will include both XX and XY cells to account for sex differences in EDC effects on sexually dimorphic brain structures.

In a first step, the chosen in vitro models and endpoints will be tested for their responsiveness to the identified relevant endocrine pathways (Table 2) with the use of endocrine model compounds, which specifically activate or inhibit the pathways of interest. Thereafter, test systems/endpoints responsive to hormonal interference will be challenged with the reference EDCs (Table 1).

To ensure a smooth transition from development to validation of the methods, the following measures are implemented throughout the project: Each participant laboratory working with human induced pluripotent stem cells (hiPSCs) will generate their own hiPSC cell bank, comprising a ‘Master cell bank’ and a ‘Working cell bank’. Furthermore, for the individual assays that are established by the partners, standard operating procedures (SOPs) for all steps of each test method will be developed from each lab and then the SOPs will be exchanged amongst the in vitro partners of the project. The goal of this lab-to-lab test method transfer is to generate a proof-of-principle that the test methods are also working in a different laboratory, a prerequisite condition of the validation process for eventual regulatory acceptance of test methods. Finally, information on the individual test methods for ED-DNT will be collected in templates recently developed for in vitro DNT test methods from the OECD211 outline [55].

The animal-based models which will be employed in the project are already part of the chemical testing guidelines recommended by the OECD and accepted internationally as standard methods for safety testing. These include rat, zebrafish, and the *Xenopus laevis* frog. The non-mammalian vertebrate models are considered advantageous alternatives to traditional in vivo DNT testing because the fundamental mechanisms underlying the development and function of the nervous system are in concordance with those in mammalian species, including humans [3]. Furthermore, these models can be used for medium to high throughput assays (including evaluation of behavioral changes) [3]. In addition, the zebrafish embryos are not considered an animal model and there is no requirement for ethical approvals associated with their use [3,42]. Within our project, the studies using the three models are designed to identify novel endpoints that are sensitive for ED-induced DNT and can be easily integrated in existing Organisation for Economic Co-operation and Development (OECD) Chemical Testing Guidelines (TGs).

Two OECD level 4 tests, TG 421 (reproductive screening test) and TG 422 (combined 28-day/reproductive screening assay), have recently been updated with peripheral endpoints for EDCs in postnatal rat offspring. However, the brains of these offspring are not analyzed, but they could be studied with molecular markers predictive of ED-induced DNT. Molecular markers for EDC effects on neurodevelopment could also increase the predictive value and specificity of the DNT module in TG 443 (level 5), where, at present, behavioral tests and neuropathological assessment do not address endocrine-related effects, and where tests of cognitive functions, one of the main EDC targets disclosed by epidemiology in humans, are not mandatory. The developmental neurotoxicity TG 426 (level 4) is also not designed to specifically identify EDC-related effects on neurodevelopment. By integrating findings between the in vitro and in vivo models from molecular (in particular omics, see below), cellular, morphological, and behavioral levels, the project will suggest a list of molecular endpoints for the amendment of existing in vivo test guidelines.

## 5. Identification of Novel “Omics” Readouts

ENDpoiNTs will generate and integrate three different types of omics data:

(i) *Transcriptomics*. Transcriptomic analyses will assess the expression pattern changes of coding and non-coding RNAs, in the context of one or several DNT endpoints as a result of EDC exposure. Transcriptomics analyses will be performed in in vitro models and in specific brain areas of exposed rats, using RNA extracted both from bulk biological material and from single cells, to dissect population-specific pattern changes. Starting from the list of top regulated genes, functional annotation and data mining will be employed to gain knowledge on the affected cellular pathways and biological processes. Further analyses of regulatory motifs and associated master regulators will allow the reconstruction of gene networks and regulatory circuits affected by the exposures.
(ii) *Epigenomics*. Epigenetic processes regulate temporal and spatial patterns of transcription and play a critical role in cell differentiation and tissue organization during development [56]. Epigenetic patterns at specific loci can change in response to environmental factors [57] and this can potentially affect health, depending on cell type and developmental stage. Epidemiological data provides increasing evidence for associations between chemical exposures and epigenetic changes [20] and an increasing number of experimental studies show that early-life exposures to EDCs and other neurotoxic compounds induce epigenetic changes, in particular DNA methylation changes, which, in some cases, have been linked to modifications in brain morphology and to adversities later in life (such as changes in anxiety-like, exploratory, and social behaviors) [20,57]. Therefore, changes in epigenetic patterns might serve as biomarkers for adverse effects on developmental processes induced by EDCs, as well as other environmental factors. In this context, ENDpoiNTs aims to assess epigenomic changes in selected in vitro and in vivo models and couple them to chemical exposures and later key events/adverse outcomes. The focus will be on DNA methylation and non-coding RNAs (including miRNA) patterns since these modifications are currently considered as the most promising epigenetic biomarkers for disease states. They are easily measured in accessible human tissue (blood, saliva, buccal epithelia) and are the most studied patterns in relation to environmental exposures [20].
(iii) *Metabolomics.* Metabolomics provides a functional readout of the physiologic state of an organism as determined by the sum of its genetic predisposition, regulation, protein abundance, and environmental influences. In ENDpoiNTs, targeted and non-targeted metabolomic approaches will be employed in exposed rats (hippocampus and MPO), zebrafish, and various in vitro models to link disruption of endocrine pathways with DNT endpoints. The aim is to understand the molecular mechanism of ED-induced DNT using exploratory and hypothesis-driven metabolomic pathway analyses, and to relate the affected molecular pathways to phenotypical, developmental, behavioral, and cognitive changes in the in vitro and in vivo assays.

Additionally, tissue-imaging mass spectrometry will be used as a powerful technique to investigate the spatial distribution of metabolites in tissue and 3D structures and to relate it to the molecular changes and functional information in rat brain tissues, cortical brain organoids, and zebrafish. This will establish the spatial bridge between toxicant exposure in organisms (brain/organoids) and metabolite changes and provide a better understanding of the pathways and mechanisms affected.

The transcriptomic, epigenomic, and metabolomic data generated will be analyzed and integrated across experimental models, exposures, and endpoints to identify:

(a) signatures that can be used as “fingerprints” for exposures with a specific endocrine mode of action;
(b) signatures that can predict certain KE and AO with relevance for DNT;
(c) panels of epigenomic, transcriptomic, and metabolomic markers which can be used to predict EDC-induced developmental neurotoxicity in both in vitro and in vivo models.

## 6. Exposure Modelling Using PBTK Models and In Silico Models (QSARs)

Within the ENDpoiNTs project, in silico screening models for identified endocrine targets will be developed to predict ED-linked DNT properties of new chemicals, and application of kinetic models will translate concentrations from the in vitro models to human exposures.

To link the active concentration metric measured in vitro with an in vivo relevant effective dose, the integration of kinetics of both systems is required, and, in this context, the use of physiologically based toxicokinetic (PBTK) modeling is considered as a key element. PBTK models can simulate concentration–time profiles of substances at a specific target site in the body, whereas with reverse dosimetry, they can be used for the prediction of external effective doses in vivo starting from the in vitro active concentrations, i.e., the presumed target doses. The quantitative in vitro to in vivo extrapolation (QIVIVE) in this project will emphasize on prenatal exposures in humans, with the focus on the fetal brain as a critical target and an oral route of exposure. All QIVIVE extrapolations will correct for different protein binding patterns between serum and test medium and operate on equivalent nominal concentrations in serum and in vitro. Presumed target effect doses will be simulated for different intake exposure scenarios (e.g., chronic vs. acute, time lags between repeated intake doses) to identify those intake patterns which lead to fetal serum concentration ranges similar to in vitro active concentrations.

To develop initial in silico predictive models for first tier screening and compound identification, state of the art machine learning and deep learning methods (random forests, support vector machines, neural networks) will be used. Various mathematical models will be developed for suspected molecular initiating events (MIEs) of importance in relation to the endocrine pathways. Receptor interactions will be based on data from public databases including, but not limited to, Tox21, ToxCast and PubChem Bioassay and the predictive models will be developed using a Mondrian conformal prediction approach to prevent information loss resulting from the highly unbalanced chemical datasets available. Similar models will be developed for prediction of blood–brain barrier penetration and brain tissue–blood partitioning, both to be used in conjunction with generic PBTK models to estimate neurotoxicity relevant exposure. The developed models will be compared to existing quantitative structure–activity relationship (QSAR) models, such as within the OECD QSAR toolbox, Danish QSAR, and VEGA, and will then be presented to OECD experts, ultimately to be reviewed by the QSAR management group.

## 7. Linking Novel Test System Results to Population-Based Human Data Using Metrics of Risk Assessment

To ensure human relevance for the developed (in vitro) endpoints, epidemiological and biomonitoring data will be used as an information source for DNT effects of EDCs. The main objective is to link human-relevant EDC exposure ranges to DNT-related effects and markers using results from the ENDpoiNTs novel test tools through risk assessment metrics, for both single compounds and chemical mixtures.

For single compounds, human-relevant levels of exposure will be linked to ED-related effects by constructing hazard quotients (HQ) per subject in multiple human studies. The HQ is the sum of each subject’s concentration of the EDC relative to the point of departure (POD) of the ED-related effect from the test system, where values above 1 are of concern. The percentage of subjects with HQ >1 will be reported per chemical and test system, thereby evaluating not only the extent of concern for human exposure, but also the sensitivity of test systems to the same chemical.

In the case of mixtures, for those subjects determined to have sufficiently similar mixtures to the mixtures tested in vitro, a ‘similar mixture risk indicator’ (SMRI; analogous to the HQ for single chemicals) will be constructed to determine the extent of the exposure relative to the POD from test systems. The calculated SMRI will be used in the analysis of epidemiology cohort studies to demonstrate the ED-related effects associated with higher index values. For comparison to the whole mixture strategy, when it is reasonable to assume additivity, a component-based strategy will be employed and a hazard index (HI) per subject from biomonitoring data will be constructed, e.g., [58].

All three metrics (i.e., the HQ, the HI, and the SMRI) and the percentage of each that exceeds 1 (indicative of level of concern) will be used to compare exposures across EU regions and over time as EDC regulatory policies change.

## 8. AOP Development, Incorporating Fundamental Neurodevelopmental Processes

A number of AOPs related to DNT and neurotoxicity are published in the AOP-Wiki, yet only two are related to an endocrine mode of action involving TH signaling (AOP 8 and 152) [59]. The main goal of ENDpoiNTs is to provide putative AOPs that function to integrate the generated and published data and to identify knowledge gaps about correlative and causative relationships between MIE, KEs, and AOs. In particular, we will link disruptions in fundamental neurodevelopmental processes (see section “Relevant models and endpoints to test ED-induced DNT”) to endocrine modes of action. The regulatory impact of constructed AOPs will be assessed using AOP assessment according to OECD principles on the basis of biological plausibility and empirical data of the MIE–KE–AO relationships [60]. Depending on their readiness, a suggestion for submission to AOP-Wiki will be made.

## 9. Conclusions and Outlook

ENDpoiNTs integrates expertise in endocrine disruption and DNT and combines state-of-the-art in silico and in vitro tools, innovative experimental designs and technologies, and advanced biostatistics on human epidemiological and biomonitoring data to generate the necessary scientific insights into the correlative and causal links between endocrine disruption and DNT. Based on these insights, it will develop in silico and in vitro tools for chemical screening and novel molecular endpoints for existing animal-based test guidelines. While we do not expect that the developed methods and endpoints will have validated European Centre for the Validation of Alternative Methods (ECVAM) or OECD TG status at the end of the five years running time of the project, we will be able to provide robust, pre-validated tools based on scientific evidence and causal relationships established within the AOP framework. This will provide a base for future projects focusing on fully validating the developed methods and endpoints.

The ultimate goal of ENDpoiNTs is to develop an integrated testing approach for ED-induced DNT and a strategy for its implementation into regulatory frameworks. The implementation strategy will be developed in the context of the EURION cluster that consists of eight sister projects advancing ED testing, and in close collaboration with the EURION international advisory panel with representatives from the OECD, the DG JRC (Joint Research Centre), and relevant European, national, and international (USA, Canada, Australia, Japan, and China) regulatory agencies. These interactions will provide regulatory guidance on the methods development in the project on the one hand, and an opportunity to continuously update regulators on the scientific findings on the other hand, which we expect will lead to faster regulatory acceptance and uptake of the developed integrated testing approach for ED-induced DNT.

## Figures and Tables

**Table 1:** List of reference EDCs (*n* = 27).

Nr.	Chemical Name	Abrev.
1	Benzyl butyl phthalate	BBzP
2	Dibutyl benzene-1,2-dicarboxylate	DBP
3	Di-ethylphthalic acid ester	DEP
4	Bis (2-ethylhexyl) phthalate	DEHP
5	Di-isodecylphthalic acid ester	DIDP
6	Di-isononyl phthalate	DINP
7	Monobutyl phthalate	MBP
8	Mono-ethylphthalic acid ester	MEP
9	Monobenzyl phthalate	MBzP
10	Mono (2-ethylhexyl)phthalate	MEHP
11	Mono-isodecylphthalic acid ester	MIDP
12	Monoisononyl phthalate	MINP
13	Bisphenol A	BPA
14	Bisphenol F	BPF
15	Perfluorohexanesulfonic acid	PFHxS
16	Perfluorooctanoic acid	PFOA
17	Perfluorooctanesulfonic acid	PFOS
18	2,2′,3,4,4′,5,5′-Heptachlorobiphenyl	PCB-180
19	3,5,6-trichloro-2-pyridinol	TCP
20	Dichlordiphenyldichlorethylen	4,4′-DDE
21	3-Phenoxybenzoic acid	3-PBA
22	Aldicarb	
23	Carbaryl	
24	Cypermethrin	
25	Endosulfan	
26	Methomyl	
27	Permethrin	

**Table 2:** List of potential endocrine targets identified as relevant for ED-induced DNT using in silico prediction.

Target Name	Chemicals	Type of Interaction
Estrogen receptor alpha and beta	BPA	Agonistic
Estradiol 17-beta-dehydrogenase 1	Endosulfan	Binding
Androgen receptor	BPA, endosulfan, 4,4′-DDE, PFHxS	Antagonistic
Thyroid hormone receptor beta	BPA, cypermethrin, endosulfan, PFHxS, permethrin	Agonistic
Glucocorticoid receptor	BPA, MEHP	Agonistic
Retinoic acid receptor alpha, beta, gamma	3-PBA, MEP, MIDP, MINP	Agonistic/not predicted
Progesterone receptor	BPA, MEHP	Not predicted
Peroxisome proliferator-activated receptor alpha, gamma, delta	Permethrin	Agonistic
Vitamin D3 receptor	Permethrin	Agonistic
Oxysterols receptor LXR-alpha	PFHxS, PFOA, PFOS	Agonistic
Prostaglandin E2 receptor EP1, EP2, EP3, EP4 subtype	MBzP	

BPA: bisphenol A, 4,4′-DDE: dichlordiphenyldichlorethylen, MBzP: monobenzyl phthalate, MEHP: mono (2-ethylhexyl) phthalate, MEP: mono-ethylphthalic acid ester, MIDP: mono-isodecylphthalic acid ester, MINP: monoisononyl phthalate, PFHxS: perfluorohexanesulfonic acid, PFOA: perfluorooctanoic acid, and PFOS: perfluorooctanesulfonic acid.

**Table 3:** ENDpoiNTs in vitro methods selected to interrogate essential DNT key events.

Assay	Endpoint	Readout	Ref
Mouse C17.2 NPC line	Proliferation	LDH release	[47]
	Differentation	bIII-tubulin staining	
	Neurite growth	Quantification of neurite length in cells stained with bIII-tubulin and DAPI	
Mouse primary NPC (SVZ)	Proliferation	Sphere size, BrdU, Ki67/PH3 stainings	[48]
	Differentiation	DCX staining	
Mouse primary neuron-oligodendrocyte co-cultures	Differentiation	MBP staining	[49]
	Myelination	MBP staining	
Rat NPCs (different brain regions)	Proliferation	Sphere Size, BrdU staining	
	Differentiation	GFAP (HCIA), O4 staining (HCIA)	
	Migration	HCIA of the bIII-tubulin or O4 stained cells which migrate from the sphere core stained with DAPI	
	Neurite growth	DAPI staining, MAP2, bIII-tubulin staining (HCIA)	
	Myelination	qRT-PCR for expression of markers of oligodendrocyte differentiation and maturation	
	Network formation and activity	Synapsin/PSD95 staining	
Rat primary cortical cultures	Network formation and activity	MEA	[50]
Human primary NPC	Proliferation	Sphere Size, BrdU staining	[42]
	Differentiation	GFAP staining (HCIA), O4 staining (HCIA), bIII-tubulin staining (HCIA)	[42]
	Migration	HCIA of the bIII-tubulin or O4 stained cells which migrate from the sphere core stained with DAPI	[42]
	Neurite growth	DAPI staining, MAP2 staining, bIII-tubulin staining	[51]
	Myelination	rtRT-PCR for expression of markers of oligodendrocyte differentiation (and maturation?)	[42]
	Network formation and activity	MEA	
Human iPSC-derived NPC (different brain regions)	Proliferation	Sphere Size, BrdU	
	Differentiation	GFAP staining	
	Migration	HCIA of the bIII-tubulin or O4 stained cells which migrate from the sphere core stained with DAPI	[42]
	Neurite growth	DAPI staining, bIII-tubulin staining	
	Network formation and activity	Synapsin/PSD95 staining, MEA	
Human iPSC-derived NCC cMINC (UKN2)	Migration	HCIA of viable cells (stained with Hoechst and calcein) which migrate to a previously unpopulated plate area	[42]
Human iPSC-derived NCC PeriTox (UKN5)	Neurite growth	HCIA of cells stained with Hoechst and calcein	[42]
Human immortalized primary NPC NeuriTox (UKN4)	Neurite growth	HCIA of cells stained with Hoechst and calcein	[42]
Human iPSC-derived neuronal co-cultures	Network formation and activity	MEA	
Human iPSC-derived cortical brain organoids	Network formation and activity	MEA	
	Molecular changes	Single cell transcriptomics, epigenomics	[52]

DAPI: 4′,6-diamidino-2-phenylindole, BrdU: bromodeoxyuridine, DCX: doublecortin, GFAP: glial fibrillary acidic protein, hiPSCs: human induced pluripotent stem cells, LDH: lactate dehydrogenase, MEA: microelectrode arrays, MAP2: microtubule-associated protein *2*, MBP: myelin basic protein, NPCs: neural progenitor cells, PSD95: postsynaptic density protein 95, and SVZ: subventricular zone.

## Abbreviations

DAPI	4′,6-diamidino-2-phenylindole
AOPs	adverse outcome pathways
AO	adverse outcome
AR	androgen receptors
BrdU	bromodeoxyuridine
CNS	central nervous system
DNT	developmental neurotoxicity
DCX	doublecortin
EDCs	endocrine-disrupting chemicals
ER	estrogen receptors
GFAP	glial fibrillary acidic protein
HI	hazard index
HQ	hazard quotients
HCIA	high content image acquisition
hiPSCs	human induced pluripotent stem cells
KE	key event
LDH	lactate dehydrogenase
LXRs	liver X/oxysterols receptors
MPO	medial preoptic area medial preoptic area
MEA	microelectrode arrays
MAP2	microtubule-associated protein 2
MIE	molecular initiating event
MBP	myelin basic protein
NPCs	neural progenitor cells
OECD	Organisation for Economic Co-operation and Development
PPARs	peroxisome proliferator-activated receptors
PBTK	physiologically based toxicokinetic
POD	point of departure
PCBs	polychlorinated biphenyls
PBDEs	polybrominated diphenyl ethers
PSD95	postsynaptic density protein 95
PR	progesterone receptor
PGE2	prostaglandin E2
QSAR	quantitative structure–activity relationship
RAR	retinoic acid receptors
RXR	retinoic X receptors
SMRI	similar mixture risk indicator
SOPs	standard operating procedures
SVZ	subventricular zone
TGs	test guidelines
TH	thyroid hormone
TR	thyroid receptors
VDR	vitamin D3 receptor
